# The role of female reproductive fluid in mediating reproductive isolation between ecotypes of the three-spined stickleback

**DOI:** 10.1186/s40851-026-00274-7

**Published:** 2026-09-22

**Authors:** Carl Smith, Eliza Szczerkowska-Majchrzak, Grzegorz Zięba

**Affiliations:** 1 Department of Ecology and Vertebrate Zoology, University of Łódź, Banacha 12/16, Łódź, 90-237 Poland; 2 Institute of Vertebrate Biology, Czech Academy of Sciences, Květná 8, Brno, 603 00 Czech Republic

**Keywords:** Cryptic female mating bias, Ecotype divergence, Gametic isolation, Ovarian fluid, Post-mating sexual selection

## Abstract

Female reproductive fluid (FRF) is increasingly recognised as a potent mediator of gametic-level cryptic female mating bias in external fertilisers, yet its role in reproductive isolation during early ecological divergence remains largely untested. We examined whether FRF from three-spined stickleback (*Gasterosteus aculeatus*) females on North Uist differentially attracts and sustains sperm from armourless (acidic loch) versus armoured (basic loch) ecotypes. Using a fully crossed diallel design across four allopatric populations and a sympatric contact zone, we quantified spermatozoa accumulation in a sperm-selection chamber and spermatozoa swimming longevity in response to conspecific and heterospecific FRF versus loch-water controls. More spermatozoa accumulated in selection chambers in response to FRF than in paired controls. Both sperm attraction and swimming longevity were enhanced by FRF. Attraction was stronger in same-ecotype pairings, whereas longevity showed clear male-environment-by-medium interactions, with acid- and basic-adapted males performing best in water chemistry matching their natal habitat. FRF therefore has the potential to function as a previously unrecognised gametic barrier, reinforcing pre-mating isolation and reducing gene flow between recently diverged ecotypes in this adaptive radiation.

**Supplementary Information:**

The online version contains supplementary material available at https://doi.org/10.1186/s40851-026-00274-7.

## Introduction

Sperm competition and cryptic female mating bias are central drivers of post-mating sexual selection [1, 2]. While the functions of seminal fluid in moderating ejaculate competitiveness are now well established [3, 4], female reproductive fluid (FRF, including ovarian fluid) has recently emerged as an additional potent mediator of sperm performance and fertilisation biases across a wide range of taxa [5–7]. In external fertilisers, FRF modulates key sperm traits, including activation, velocity, motility, longevity, straightness of trajectory, and chemoattraction, which can bias paternity towards preferred or genetically compatible males [8–10]. In Chinook salmon, variation in the ionic composition of ovarian fluid, particularly Ca^2 +^ and Mg^2 +^, has been shown to differentially affect sperm longevity and motility among males, providing a chemical basis for cryptic female choice [11]. Ovarian fluid also differentially enhances sperm velocity among males in this species [12], and these effects translate into higher fertilisation success and embryo survival under sperm competition [13]. Concentration-dependent effects of FRF influence the relative performance of alternative male phenotypes in the grass goby [14], while in the guppy FRF mediates cryptic preference for genetically unrelated males [15]. Recent studies using sperm selection chambers have demonstrated that FRF can exert both inter- and intra-ejaculate selection. For instance, in zebrafish, FRF preferentially attracts more numerous, viable, and higher-quality sperm (with greater DNA integrity) from within the same ejaculate [10]. In salmonids, conspecific ovarian fluid doubles sperm motile lifespan, straightens swimming trajectories, and selectively attracts conspecific sperm, thereby promoting assortative fertilisation and reproductive isolation [7]. In the three-spined stickleback, FRF has similarly been shown to extend the longevity of sperm activity and thereby fertilisation success [16, 17]. Comparable patterns of differential chemotaxis that moderate sperm competition have been documented in broadcast-spawning marine invertebrates, where egg-derived chemoattractants bias fertilisation success towards genetically compatible males [9]. These findings, together with experimental evidence that FRF can shift the outcome of sperm competition in other externally fertilising fish [8], underscore FRF as a powerful mechanism of gametic-level cryptic female mating bias even in the absence of internal fertilisation. Importantly, although FRF is female-derived and can bias fertilisation outcomes, it acts mechanistically as a gametic environment rather than an active, context-dependent female decision during fertilisation.

Despite this growing body of work, including demonstrations that FRF mediates cryptic female choice and conspecific sperm precedence in Atlantic [7] and Chinook salmon [12, 13], and differential sperm attraction in zebrafish [10], the role of FRF in the evolution of reproductive barriers during early ecological divergence within a species remains largely unexplored. Thus, while evidence shows that FRF can bias sperm performance and fertilisation outcomes, it remains unclear whether such effects operate between recently diverged ecotypes and can contribute to assortative fertilisation prior to the evolution of post-zygotic isolation.

The three-spined stickleback (*Gasterosteus aculeatus*) populations on North Uist, Scottish Outer Hebrides, provide an outstanding natural system for testing this question. These populations have undergone a recent adaptive radiation into distinct ecotypes associated with contrasting environments: armour-reduced (‘armourless’) forms in acidic, peat-stained lochs and low-plated (‘armoured’) forms in calcium-rich machair lochs [18–20]. Genomic studies reveal striking parallelism in adaptation to basic versus acidic habitats, driven by predictable sorting of standing genetic variation from a marine ancestor [18]. Although secondary contact and limited hybridisation occur in some lochs [19], previous experimental work has demonstrated strong pre-mating sexual isolation between these ecotypes, driven in part by male traits [20]. In vitro fertilisations have also revealed no post-zygotic incompatibilities or ecotype-specific fertilisation biases [20].

Whether additional post-mating, pre-zygotic barriers operate at the gametic level, mediated by differential responses of sperm to FRF, remains unexamined. Given the pronounced ecological, phenotypic, and genomic divergence between ecotypes and the potential for gene flow in contact zones [19], FRF could reinforce reproductive isolation by differentially attracting sperm or enhancing sperm performance according to male–female ecotype or population of origin, potentially reflecting adaptation of sperm performance to local environmental conditions, such as water chemistry. Here we test this hypothesis. Using a sperm selection chamber [6], we quantified sperm accumulation in FRF versus controls across a fully crossed diallel design that included all possible male–female combinations from four allopatric populations (two armourless and two armoured) and one sympatric contact-zone population, thereby disentangling the effects of ecotype from those of population of origin. In parallel, we assessed the influence of FRF on sperm swimming longevity against a set of alternative controls. If FRF contributes to assortative fertilisation, we predicted greater spermatozoa accumulation and enhanced longevity in same-population or ecotype FRF. Thus, this study addresses the potential role of FRF as a mechanism that maintains divergence during the early stages of ecological speciation.

## Materials and methods

### Sampling sites, fish collection and husbandry

**Fig. 1:**
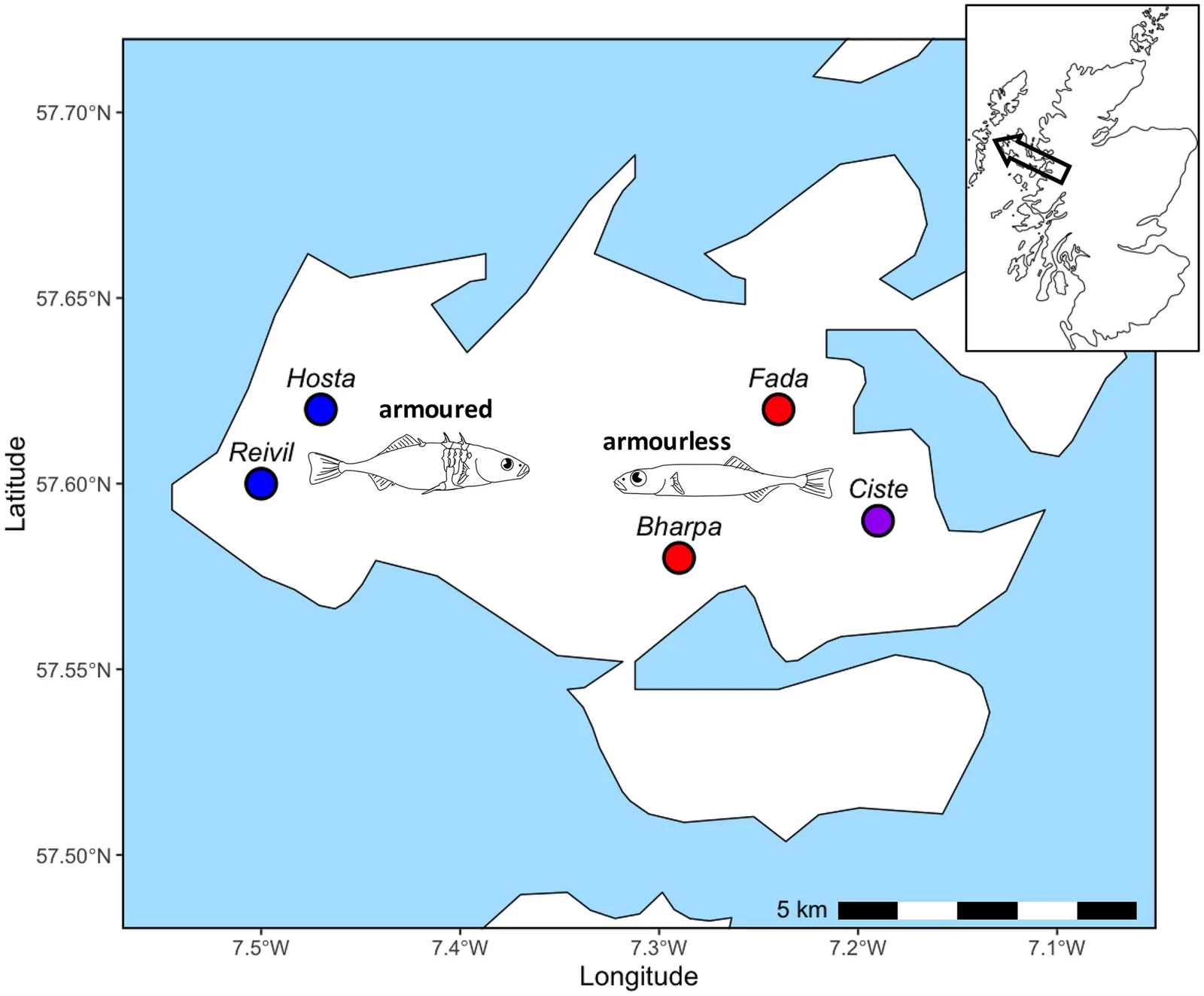
Map of the study area on North Uist, outer Hebrides, Scotland, showing the five sampling locations. Blue symbols indicate armoured ecotype populations from basic (*machair*) lochs; red symbols indicate armourless ecotype populations from acidic (peat-stained) lochs; the purple symbol indicates the sympatric contact zone at Loch Ciste. Inset shows the location of North Uist within Scotland

Three-spined sticklebacks were collected with hand nets from five populations on North Uist in May 2026 (Fig. 1). To disentangle ecotype-specific effects from those of the population of origin, two populations (Lochs Fada and Bharpa) comprised an armour-reduced ecotype from peat-stained, low-pH lochs (pH 6.5 and 6.4, respectively; hereafter termed ‘armourless’), and the other two (Lochs Reivil and Hosta) comprised low-plated ecotypes (‘armoured’) from calcium-rich *machair* lochs (pH 8.1 and 8.2, respectively; Table 1, Fig. 1). Ecotypes were assigned at capture by phenotype and confirmed by lateral plate counts (armourless: 0–2 plates; armoured: ≥3 plates). These populations are unconnected, and the populations within them are genetically discrete [18]. Fish were also collected from a fifth site, Loch Ciste, where the armourless and armoured ecotypes co-occur through secondary contact. This unusual situation arises because an acidic peat loch, Loch Geireann, debouches into basic freshwater Loch Ciste. Loch Geireann supports a population of the armourless ecotype, some of which escape into Loch Ciste through an artificial culvert under a road, where they are confined to a low pH pool at the Loch’s western end. Dean et al. (2024) [19] conducted a genomic analysis of these populations, demonstrating that the resident freshwater armoured ecotype and introduced armourless ecotype show no evidence of introgression. In Loch Ciste, armourless ecotype fish were collected from the low-pH pool at the western end of the loch (pH 6.4; originating from Loch Geireann); armoured ecotype fish were collected from the main body of the loch (pH 7.1).

**Table 1:** Populations of three-spined sticklebacks used in the study

Population	Map reference	Ecotype	Loch type	pH	*Area (km*^*2*^)
Loch Bharpa	57.572812, −7.295417	armourless	acidic	6.4	0.53
Loch Fada	57.614524, −7.223573	armourless	acidic	6.5	1.93
Ciste western pool	57.599015, −7.180305	armourless	acidic	6.4	0.02
Ciste main body	57.598425, −7.177216	armoured	basic	7.1	0.02
Loch Hosta	57.627345, −7.490477	armoured	basic	8.2	0.33
Loch Reivil	57.611216, −7.513549	armoured	basic	8.1	0.06

An initial collection of twenty males and females from each population was captured and transported in loch water to an aquarium facility at ambient temperature (13–15 °C) under natural daylight. In Loch Ciste, armourless ecotype fish were collected from the low-pH western pool (pH 6.4; originating from Loch Geireann); armoured ecotype fish from the main body of the loch (pH 7.1). Ecotypes were assigned at capture based on phenotype and subsequently via lateral plate counts. Water samples from each site were collected to measure pH, salinity and absorbance. The two sexes of each population were housed separately in aquaria measuring 500 mm (length) ×350 mm (width) ×250 mm (depth). The aquaria were continuously aerated, filtered, and furnished with vegetation to serve as refuges [21]. Each aquarium contained a 5 mm layer of washed sand from the loch of origin and was filled with 40 litres of water from the same loch. Fish were fed three times daily to satiation with bloodworm (*Chironomus* spp.) and live zooplankton. Water quality in aquaria and tanks was monitored for ammonia, nitrite and nitrate using aquarium test kits (NT Labs, Tonbridge, UK). Dissolved oxygen was not measured, as continuous aeration readily maintains near-saturation levels (>8 mg l^−1^) at the low stocking densities used [22]. Additional collections of small numbers (1–3) of females in spawning condition were made on a further four occasions from specific lochs (Reivil, Hosta, Fada, Ciste) to ensure a balanced experimental design.

### Experimental design and protocol

Our aim was to separate the effects of ecotype from those of the population of origin on FRF function. To achieve this, we first conducted a fully crossed diallel design using the four allopatric populations: two armourless populations from acidic lochs (Lochs Bharpa and Fada) and two armoured populations from basic lochs (Lochs Hosta and Reivil). This produced all 16 possible male–female population combinations. Each combination was tested in three independent temporal replicates conducted over 21 days in May 2026, yielding 48 trials.

In parallel, we sampled both armourless and armoured ecotypes that co-occur in a rare sympatric contact zone in Loch Ciste. For these co-occurring ecotypes, we conducted an additional 24 trials, including within-ecotype and between-ecotype (mixed) pairings. These trials enabled separate models to test local adaptation and reproductive isolation within the contact zone. The overall sample size was therefore *N* = 72 (Table S1).

Gravid females of a predetermined population and ecotype, identified visually by clearly discernible abdominal swelling and dilation of the genital opening, were anaesthetised in MS222 and placed on a dry glass Petri dish. Eggs and accompanying FRF were stripped by applying gentle pressure to the abdomen. Clutch size was recorded for each female. A volume of distilled water equivalent to 4 μl/egg was added to the clutch. The eggs were carefully rinsed, and two 100 μl aliquots of the resulting diluted FRF were transferred to separate 1 ml Eppendorf tubes. The resulting diluted aliquot was estimated to contain approximately 1–5% FRF by volume. This estimate is based on residual fluid volumes of ~0.5–2 µl per egg, the addition of 4 µl distilled water per egg, and the further dilution that occurs during rinsing; the final concentration therefore falls substantially below the pre-rinse range of ~8–33%. The estimated 1–5% range overlaps the 0.75–3% ovarian-fluid concentrations previously shown by Elofsson et al. (2006) [23] to extend stickleback sperm motility from ~1 min in freshwater to several hours, indicating that the assay conditions are within an ecologically relevant range. Differences in FRF pH and ionic composition between acid- and basic-ecotype females were not measured in the present study; these parameters are known to influence sperm performance and should be prioritised in future work. One aliquot was subsequently used for sperm attraction assays and the other for sperm swimming longevity. Females were transferred to an aquarium to recover, were not used again in the study, and were subsequently released at the site from which they were collected.

A male from a predetermined population and ecotype was euthanised with an MS-222 overdose, followed by spinal section. The testes were dissected and transferred to a 35 mm glass Petri dish containing 1 ml of teleost saline (0.9% NaCl), then disrupted with a dissecting needle to release spermatozoa. After gentle mixing, a 0.5 ml sample of the sperm solution was transferred to a 1 ml Eppendorf tube. Spermatozoa in the solution were counted using a haemocytometer and included as a covariate in statistical models. Sperm and FRF solutions were always used within 15 min of collection; pilot tests showed that spermatozoa remained motile well beyond this time window. Final sample sizes for the male–female combination are given in Table S1. Unused males were released at the site from which they were collected.

### Sperm attraction assays

Sperm attraction procedures followed the protocol described by Devigili et al. (2021) [6]. We used a 3D-printed sperm selection chamber with a central activation well for sperm samples and two lateral wells for FRF (treatment) and loch water (control). To expel air from the chamber, 10 μl of distilled water was loaded into each well. A 5 μl sample of FRF and water from the male’s loch of origin was then loaded into the lateral wells according to a random, predetermined schedule. The liquids were allowed to form a gradient in the central well for 90 seconds, after which 10 μl of sperm solution was loaded into the central activation well. After 90 seconds, 5 μl of liquid was collected from the lateral wells and transferred to a Neubauer improved haemocytometer, and a count of spermatozoa was made for each of four corner sets of 16 squares at x400 magnification. Spermatozoa were counted only if they sat within a square or on the right-hand or bottom boundary line. Spermatozoa concentration was calculated by averaging the spermatozoa count across all four sets of corner squares. This figure was multiplied by 10 to give an average spermatozoa concentration per 1 μl for FRF and loch water control. A spermatozoa count was similarly conducted for the sperm stock solution as a covariate in the analysis, and expressed per 1 μl. Sperm selection chambers were washed between trials by flushing with distilled water. Two sperm selection chambers were used and were randomly assigned among trials.

This assay quantifies differential sperm accumulation in the lateral wells in response to FRF versus control medium. This pattern is consistent with chemoattraction [6] but does not constitute definitive proof of molecular chemotaxis, as local gradients in FRF could also influence sperm swimming persistence or trajectory.

### Spermatozoa swimming longevity

Sperm swimming longevity was measured by introducing a series of media into a Neubauer improved haemocytometer using a pipette. An insulin syringe needle was then dipped into the stored sperm solution, and spermatozoa were introduced under the coverslip. Spermatozoa swimming longevity was measured as the time from activation until all spermatozoa ceased active swimming; movement without progress was not considered active swimming. Sperm longevity assessments were conducted by a single observer who was not blind to male–female ecotype, population of origin, or test medium. Repeatability of the visual endpoint (cessation of all forward movement) was not formally quantified. These limitations are acknowledged; future studies using computer-assisted sperm analysis (CASA) would allow objective measurement of progressive motility and velocity decline and remove potential observer bias.

A single trial was conducted for each experimental pairing. The media used to test sperm swimming longevity were: experimental FRF; FRF collected from nine-spined sticklebacks (*Pungitius pungitius*); water from basic lochs Hosta and Reivil; water from acid lochs Bharpa and Fada; water from two unnamed acidic fishless lochs (Loch K 57.591779, −7.222312, pH 6.2 and Loch G 57.592705, −7.217424, pH 5.0); and finally, distilled water. Nine-spined stickleback FRF in each trial was obtained from gravid females from Loch Hosta (where both species co-occur) using an identical extraction protocol to that for three-spined females and served as a heterospecific control, with a single female used for each trial. Total time from testis disruption to final medium test was < 45 min. Pilot tests confirmed > 90% motility retention across this time window. The order of testing was randomly predetermined and balanced. For tests of swimming longevity in armoured and armourless males from Loch Ciste, two additional media were tested: loch water from the western end of the Loch (pH 6.4) and from its eastern end (pH 7.1).

### Data analysis

All statistical analyses were conducted in R version 4.6.1 [24] using RStudio. Data exploration followed the protocol of Zuur & Ieno (2016) [25] and included visual inspection for outliers, assessment of normality and homogeneity of variances, and evaluation of collinearity among predictors using the *GGally* package [26]. Model diagnostics were performed with the *DHARMa* package [27] by simulating scaled residuals with inspection of QQ plots, residual-versus-fitted-value plots, and covariate-specific residual plots, and by testing for uniformity and outliers. Overdispersion was assessed with the *performance* package [28]. Šidák post-hoc pairwise contrasts were performed with *emmeans* [29].

For sperm attraction assays, the response variable was the number of spermatozoa counted in samples taken from each lateral well of the sperm selection chamber. Because counts were non-negative integers and overdispersed, we fitted generalised linear mixed models (GLMMs) with a negative binomial error distribution and a log link using the *glmmTMB* package [30]. The full model included log sperm stock concentration and air temperature as covariates, test medium (FRF versus loch-water control), male-female ecotype match (same versus different), male-female population match (same versus different), and two-way interactions between medium and both ecotype match and population match to test for assortative gametic responses. Additional covariates included clutch size, female SL, and male SL. Clutch size and female SL were strongly collinear, and male SL was weakly collinear with stock concentration. These covariates were not included in the final model; when included, none were significant. To control for any potential bias in sperm chamber construction, lateral wells (right and left) and sperm chambers (1 and 2) were also included in an initial model, though neither proved to impose any significant bias and were similarly dropped from the final model. Male-female pair identity was included as a random intercept to account for the paired design and repeated-measures structure. Incidence rate ratios (IRRs) and 95% confidence intervals were obtained by exponentiating the model coefficients. An IRR greater than 1 indicates an increase in the response relative to the reference level, while an IRR less than 1 indicates a decrease; for example, an IRR of 2 corresponds to a doubling (100% increase) of the expected count or duration. Post hoc contrasts were not required, as interactions were interpreted directly from the full model. Effect sizes are reported as marginal and conditional R^2^ following Nakagawa et al. (2017) [31].

Spermatozoa swimming longevity (time in seconds until all spermatozoa ceased active forward movement) was also analysed using negative binomial generalised linear mixed models (GLMMs) with a log link. For the full dataset, fixed effects included scaled sperm stock concentration, temperature, ecotype match, male environment of origin (acid vs. basic), and test medium, a categorical factor with six levels: 3-spined FRF, 9-spined FRF, mean of basic lochs (Hosta and Reivil), mean of acid lochs (Bharpa and Fada), mean of fishless lochs (G and K), and distilled water, plus the interaction between male environment of origin and test medium. Male-female pair identity was included as a random intercept. For the sympatric contact-zone fish from Loch Ciste, a separate negative binomial GLMM was fitted with the same structure but with an expanded medium factor that included acidic water collected from the low-pH pool at the Loch’s western end and basic-pH water from the main body of the Loch. Šidák post hoc pairwise contrasts were performed with the *emmeans* package [29] to identify which pairwise media differed from each other. We also fitted a separate sub-model for acid versus basic loch water only (excluding Ciste fish) and a separate model focused exclusively on the two Ciste water types (acid vs. basic). These models were used to identify environment-specific effects of male origin (acid vs. basic males) on spermatozoa swimming longevity and to test for potential local adaptation or habitat-by-ecotype interactions in spermatozoa motility.

The effects of male and female body size, female clutch size, and, in the sperm attraction assay, the sperm chamber and the lateral wells used (right and left) were included in all initial models. These covariates showed no detectable effects on the response (small, non-significant coefficients) and did not alleviate collinearity issues among retained predictors; they were therefore removed during backward model selection using the ‘drop1’ function in base R. Temperature was retained in all models to account for day-to-day variation. Results for the full fitted models are presented in Supplementary Tables S2–S5. In each case, model fit is poorer (based on AIC), but the results are qualitatively identical to the refined models.

## Results

### Sperm attraction assays

Spermatozoa accumulated significantly more in chambers containing female reproductive fluid (FRF) than in paired loch-water controls (Table 2). The negative binomial GLMM showed a strong main effect of medium (FRF vs. control: IRR = 2.22, *p* < 0.001), indicating that FRF attracted more than twice as many spermatozoa on average.

**Table 2:** Negative binomial GLMM of spermatozoa count as a function of test medium, pair ecotype and population, and temperature. Sperm stock solution was included as a covariate. Dispersion ratio = 1.58 (*p* = 0.096). Model random effects: σ^2^ = 0.01; ICC = 0.94, τ_00 Pair_ = 0.13, N _pair_ = 72. Marginal R^2^ = 0.74, conditional R^2^ = 0.98, AIC = 2106. IRR is the incidence rate ratio

Coefficient	Estimate	95% CI	IRR	95% CI	P
Intercept	0.69	−1.84–3.22	2.00	67–3654	0.592
Stock count	0.58	0.41–0.76	1.79	1.51–2.13	**<0.001**
Medium_(FRF)_	0.80	0.55–1.04	2.22	1.73–2.84	**<0.001**
Ecotype_(same)_	−0.11	−0.43–0.22	0.90	0.65–1.24	0.512
Population_(same)_	0.14	−0.18–0.46	1.15	0.83–1.59	0.391
Temperature	−0.01	−0.12–0.12	0.99	0.89–1.12	0.977
Medium_(FRF)_:Ecotype_(same)_	0.50	0.18–0.82	1.65	1.20–2.26	**0.002**
Medium_(FRF)_:Population _(same)_	−0.19	−0.50–0.13	0.83	0.60–1.14	0.250

**Fig. 2:**
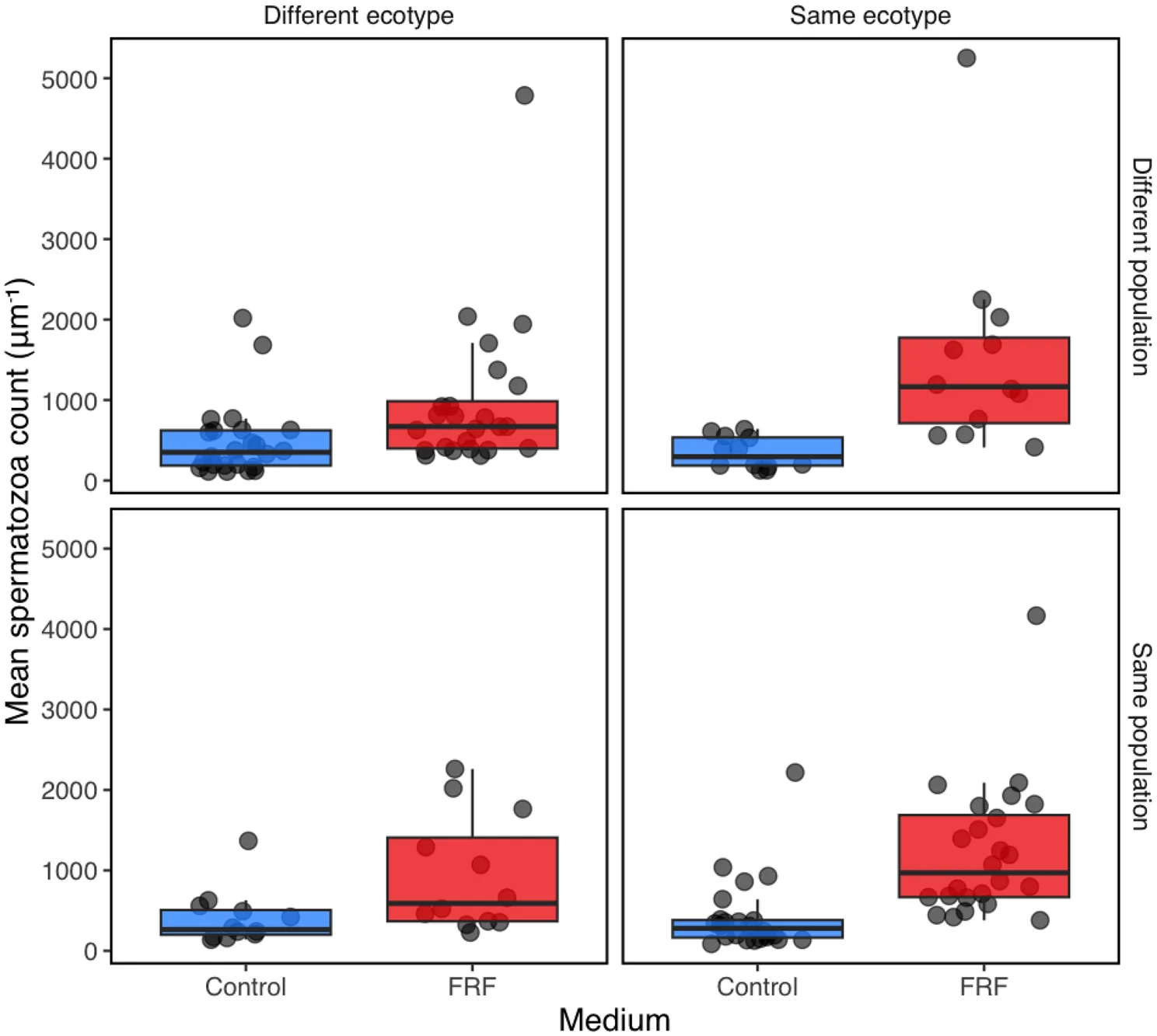
Spermatozoa accumulation (mean spermatozoa count per μl) in female reproductive fluid (FRF, red) versus paired loch-water controls (blue) in sperm selection chambers. Results are shown for four combinations of population match and ecotype match. Boxes show medians and interquartile ranges; whiskers extend to 1.5 × IQR; points are individual trials. Data from three replicates of a fully crossed diallel design plus loch Ciste pairings (*N* = 72 trials)

A significant interaction between medium and ecotype match was detected (IRR = 1.65, *p* = 0.002), demonstrating assortative gametic attraction. Sperm from males of the same ecotype as the female showed stronger (65% greater) accumulation in FRF compared with different-ecotype pairings. No significant interaction was found between medium and population match (IRR = 0.83, *p* = 0.251), indicating that the assortative response was driven primarily by ecotype rather than by population of origin. Sperm stock concentration was a strong positive predictor of counts in both wells (IRR = 3.89, *p* < 0.001). These patterns are illustrated in Fig. 2, which shows consistently higher sperm counts in FRF (red) than in control (blue) wells, with the strongest preference evident in same-ecotype combinations.

### Spermatozoa swimming longevity

**Fig. 3:**
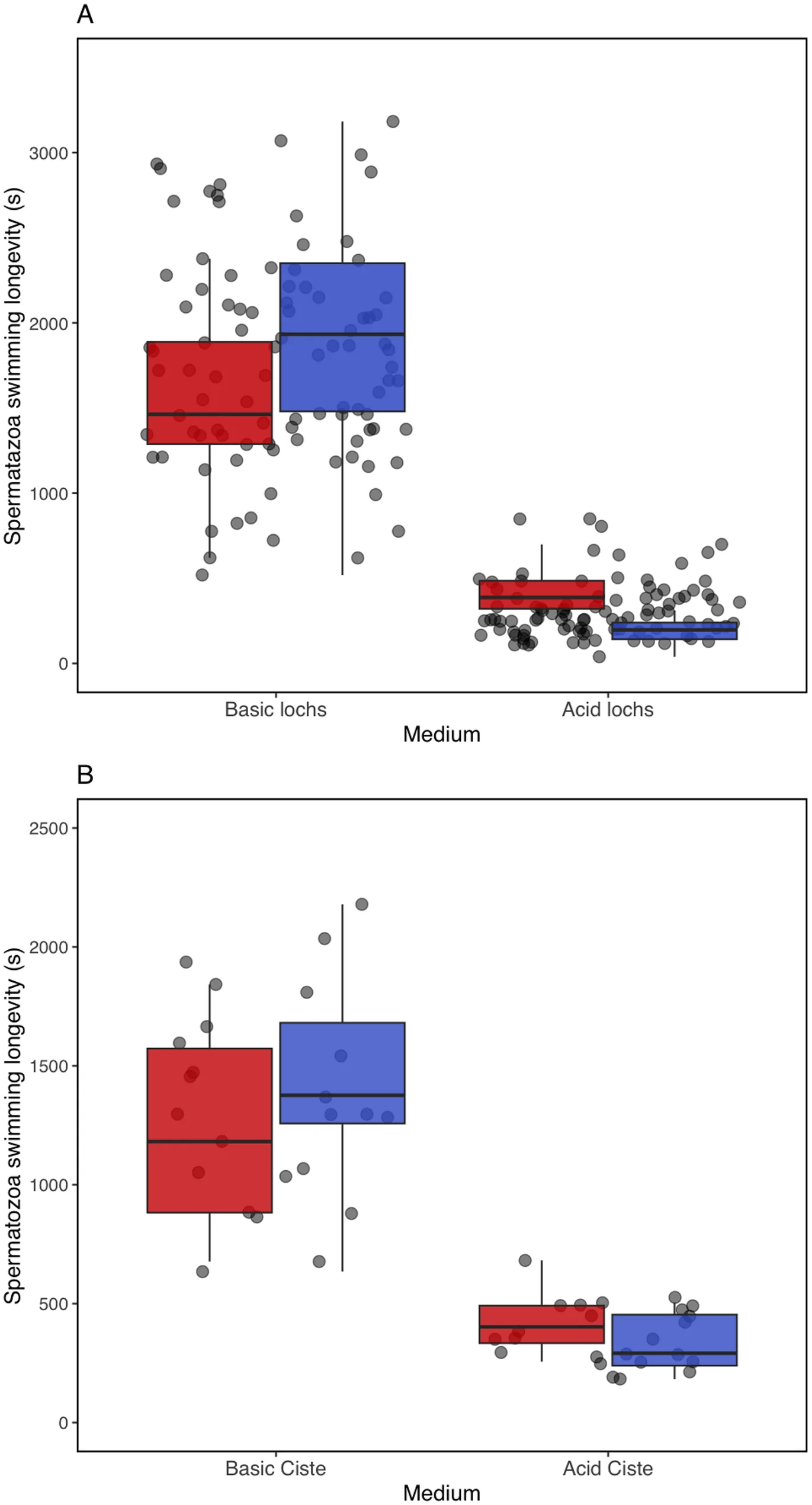
Sperm swimming longevity in: (**A**) acid versus basic loch water (excluding Ciste fish) and (**B**) acid versus basic water from the sympatric contact zone in loch Ciste for acid (red) and basic (blue) adapted males. Boxes show medians and interquartile ranges; whiskers extend to 1.5 × IQR; points are individual trials

Sperm swimming longevity was strongly influenced by the activation medium (Table 3). The longest motility durations were recorded in three-spined stickleback FRF, followed by nine-spined FRF, with progressively shorter durations in basic loch water, acid loch water, fishless loch water, and distilled water (Šidák post hoc contrasts, all relevant comparisons *p* < 0.001 except fishless vs. distilled water, *p* = 0.999). A significant male environment × medium interaction was present (Table 3), indicating environment-specific responses. Basic males maintained relatively longer sperm longevity in basic loch water, whereas acid males performed relatively better in acid loch water (Table 4). A focused analysis of acid versus basic loch water confirmed this pattern, with a significant male environment × medium interaction (Table 5; IRR = 0.38, *p* < 0.001). The spermatozoa of males from basic lochs showed markedly (62%) reduced swimming longevity in acid water but were extended in basic loch water (Fig. 3A).

**Table 3:** Negative binomial GLMM of spermatozoa motility duration as a function of test medium, temperature, pair ecotype, and male environment of origin. Sperm stock solution was included as a covariate. Dispersion ratio = 0.95 (*p* = 0.896). Model random effects: σ^2^ = 0.02; ICC = 0.80, τ_00 Pair_ = 0.07, N _pair_ = 72. Marginal R^2^ = 0.95, conditional R^2^ = 0.99, AIC = 8479. IRR is the incidence rate ratio. Pairwise Šidák post-hoc contrasts showed highly significant differences (*p* < 0.001) in spermatozoa longevity among media in the order: 3-spine FRF > 9-spine FRF > basic loch water > acid loch water > fishless loch water/distilled water. There was no significant difference in longevity between fishless loch water and distilled water (*p* = 0.999)

Coefficient	Estimate	95% CI	IRR	95% CI	P
Intercept	9.35	7.99–10.71	11470	2943– 44,698	**<0.001**
Log_10_ stock count	−0.16	−0.27 – −0.04	0.85	0.76–0.96	**0.007**
Temperature	−0.03	−0.10–0.04	0.97	0.90–1.04	0.356
Ecotype_(same)_	0.06	−0.07–0.19	1.06	0.94–1.21	0.348
Male environment_(basic)_	0.12	−0.03–0.28	1.13	0.97–1.32	0.114
Medium_(9-spine FRF)_	−0.11	−0.18 – −0.04	0.89	0.83–0.96	**0.002**
Medium_(basic lochs)_	−0.54	−0.61 – −0.47	0.58	0.54–0.62	**<0.001**
Medium_(acid lochs)_	−1.88	−1.97 – −1.78	0.15	0.14–0.17	**<0.001**
Medium_(fishless lochs)_	−3.00	−3.15 – −2.85	0.05	0.04–0.06	**<0.001**
Medium_(distilled water)_	−3.09	−3.30 – −2.85	0.05	0.04–0.06	**<0.001**
Male environment_(basic)_: Medium_(9-spine FRF)_	0.01	−0.09–0.11	1.01	0.91–1.12	0.803
Male environment_(basic)_: Medium_(basic lochs)_	0.17	0.08–0.27	1.19	1.09–1.31	**<0.001**
Male environment_(basic)_: Medium_(acid lochs)_	−0.63	−0.79 – −0.48	0.53	0.45–0.62	**<0.001**
Male environment_(basic)_: Medium_(fishless lochs)_	−0.28	−0.51 – −0.09	0.75	0.60–0.93	**0.010**
Male environment_(basic)_: Medium_(distilled water)_	0.02	−0.27–0.32	1.02	0.76–1.37	0.883

**Table 4:** Šidák post-hoc pairwise contrasts of spermatozoa swimming longevity among activation media from the negative binomial GLMM (Table 3).

Contrast	Ratio	P
3-spined FRF − 9-spined FRF	1.01	0.999
3-spined FRF – Basic loch water	1.19	**0.004**
3-spined FRF – Acid loch water	0.53	**<0.001**
3-spined FRF – Fishless loch water	0.75	0.140
3-spined FRF – Distilled water	1.02	0.999
9-spined FRF – Basic loch water	1.18	**0.015**
9-spined FRF – Acid loch water	0.52	**<0.001**
9-spined FRF – Fishless loch water	0.74	0.107
9-spined FRF – Distilled water	1.01	0.999
Basic loch water – Acid loch water	0.45	**<0.001**
Basic loch water – Fishless loch water	0.63	**0.001**
Basic loch water – Distilled water	0.86	0.996
Acid loch water – Fishless loch water	1.41	0.101
Acid loch water – Distilled water	1.93	**0.001**
Fishless loch water – Distilled water	1.36	0.738

**Table 5:** Negative binomial GLMM of spermatozoa motility duration as a function of test medium, temperature, pair ecotype, and male environment of origin for acid and basic loch water only. Sperm stock solution was included as a covariate. Dispersion ratio = 1.00 (*p* = 0.872). Model random effects: σ^2^ = 0.03; ICC = 0.73, τ_00 Pair_ = 0.08, N _pair_ = 48. Marginal R^2^ = 0.90, conditional R^2^ = 0.97, AIC = 2510. IRR is the incidence rate ratio

Coefficient	Estimate	95% CI	IRR	95% CI	P
Intercept	9.20	7.50–10.90	9891	1809– 54,086	**<0.001**
Log_10_ stock count	−0.12	−0.26–0.02	0.85	0.77–1.02	0.096
Temperature	−0.08	−0.17–0.01	0.93	0.85–1.01	0.089
Ecotype_(same)_	−0.01	−0.18–0.16	0.99	0.84–1.18	0.919
Male environment_(basic)_	0.25	0.06–0.44	1.28	1.06–1.55	**0.011**
Medium_(acid lochs)_	−1.30	−1.39 – −1.21	0.27	0.25–0.30	**<0.001**
Male environment_(basic)_: Medium_(acid lochs)_	−0.97	−1.13 – −0.81	0.38	0.32–0.44	**<0.001**

**Fig. 4:**
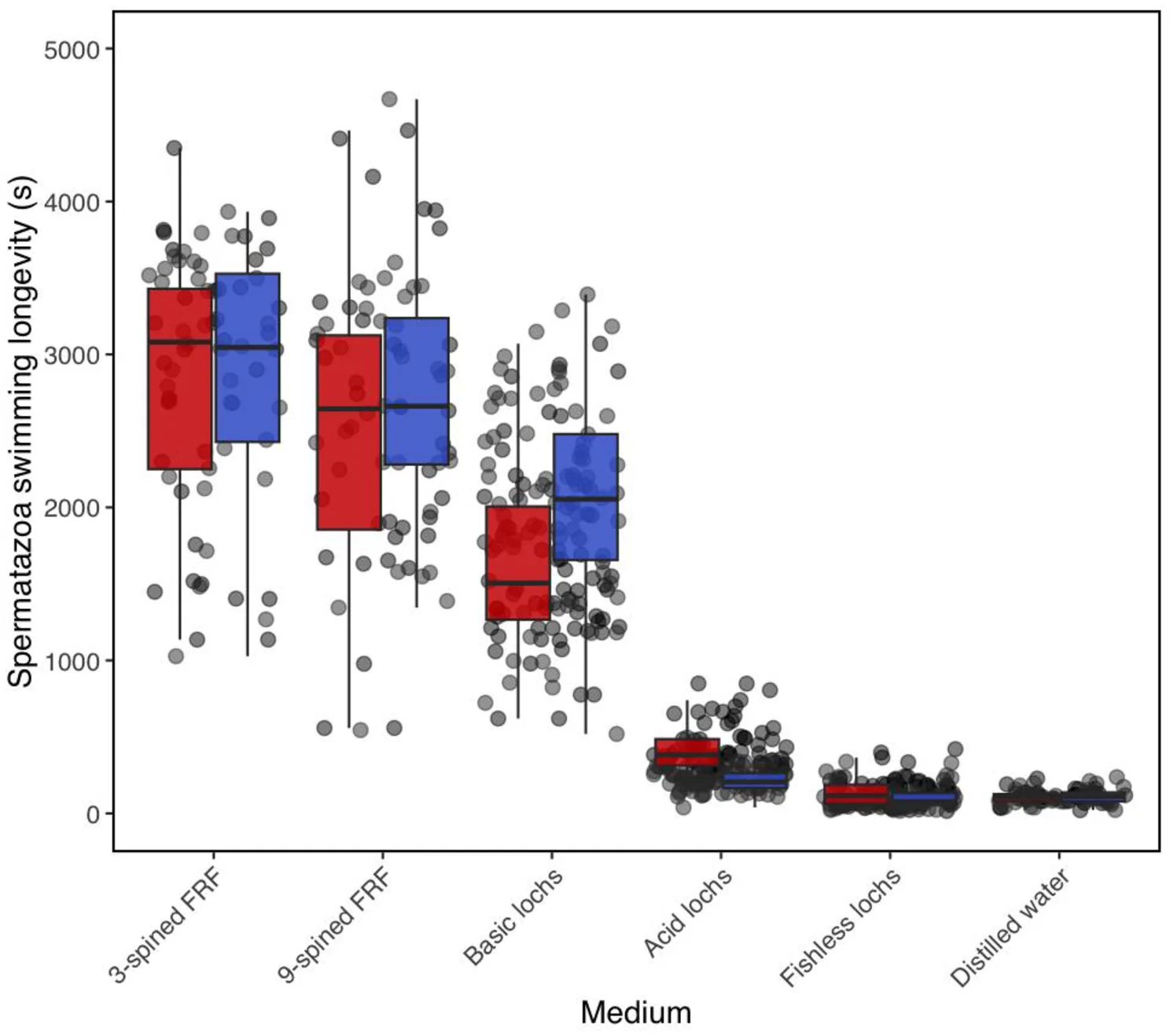
Spermatozoa swimming longevity (seconds) across activation media for acid (red) and basic (blue) adapted males. Boxes show medians and interquartile ranges; whiskers extend to 1.5 × IQR; points are individual trials. Media ordered by overall performance: three-spined FRF, nine-spined FRF, basic loch water, acid loch water, fishless loch water, and distilled water (see Table 4 for pairwise *post-hoc* contrasts)

In the sympatric contact zone (Loch Ciste), a similar interaction between male environment and local water type was observed (Table 6). Spermatozoa from armoured, basic-adapted Ciste males performed better in basic Ciste water (Table 6; IRR = 0.67, *p* = 0.012), corresponding to an approximately 33% reduction in longevity in the mismatched (acidic) medium, whereas acid-adapted Ciste males performed better in acidic Ciste water (Fig. 3B). Overall, FRF consistently prolonged sperm longevity more than any loch water or distilled water control (Fig. 4), supporting a role for FRF in enhancing gamete performance.

**Table 6:** Negative binomial GLMM of spermatozoa motility duration as a function of test medium, temperature, pair ecotype, and male environment of origin for acid and basic water for Loch Ciste only. Sperm stock solution was included as a covariate. Dispersion ratio = 1.12 (*p* = 0.552). Model random effects: σ^2^ = 0.02; ICC = 0.59, τ_00 Pair_ = 0.03, N _pair_ = 24. Marginal R^2^ = 0.88, conditional R^2^ = 0.95, AIC = 669. IRR is the incidence rate ratio

Coefficient	Estimate	95% CI	IRR	95% CI	P
Intercept	5.47	2.99–7.95	237	20–2832	**<0.001**
Log_10_ stock count	0.14	−0.09–0.37	1.15	0.92–1.45	0.223
Temperature	0.05	−0.07–0.17	1.05	0.93–1.19	0.438
Ecotype_(same)_	0.11	−0.09–0.31	1.12	0.92–1.36	0.266
Male environment_(basic)_	0.08	−0.16–0.32	1.09	0.85–1.38	0.500
Medium_(acid water)_	−1.08	−1.29 – −0.87	0.34	0.28–0.42	**<0.001**
Male environment_(basic)_: Medium_(acid water)_	−0.39	−0.70– −0.09	0.67	0.50–0.92	**0.012**

## Discussion

In this study, we demonstrate that female reproductive fluid (FRF) from North Uist three-spined stickleback females differentially attracts and supports spermatozoa in an ecotype-assortative manner. Using a fully crossed diallel design spanning allopatric and sympatric populations, we show that spermatozoa accumulate more strongly in FRF than in loch-water controls, and that this effect is significantly stronger in same-ecotype pairings. Spermatozoa swimming longevity was greatest in conspecific FRF and showed clear male environment-by-medium interactions, with acid- and basic-adapted males performing best in water chemistry matching their natal habitat. These post-mating, pre-zygotic effects indicate that FRF acts as a cryptic gametic filter, reinforcing reproductive isolation between recently diverged ecotypes.

Our finding of ecotype-assortative sperm attraction directly addresses a key knowledge gap: the potential for FRF-mediated gametic isolation during early ecological speciation. The significant medium × ecotype interaction (Table 2, Fig. 2) illustrates that FRF exerts stronger chemoattraction towards spermatozoa from males of the same ecotype, irrespective of population of origin. This pattern aligns with studies in salmonids [7] and zebrafish [10], where FRF biases sperm accumulation towards compatible or high-quality gametes, but extends these observations to incipient ecotype divergence within a single species. The absence of a comparable population-level interaction suggests that selection on FRF function has acted primarily at the ecological (acid vs. basic) rather than the fine-scale population level, consistent with the strong parallel genomic divergence reported between these habitats [18, 19].

Spermatozoa swimming longevity results reinforce the role of FRF as a mechanism of cryptic female mating bias. Conspecific FRF produced markedly longer motility durations than heterospecific FRF or any environmental water, confirming and extending earlier work on sticklebacks [16, 17]. The pronounced male environment × medium interaction demonstrates local adaptation at the gametic level: basic males sustained longer spermatozoa motility in basic water, whereas acid males performed better in acid water. This pattern was replicated in the sympatric contact zone in Loch Ciste, where males showed superior performance in water chemistry matching their respective loch sections of origin. Such environment-specific effects suggest that FRF and local water chemistry may interact to enhance fertilisation success under natural spawning conditions, thereby providing a post-mating advantage to locally adapted males and potentially limiting hybridisation in secondary contact zones. These findings extend established evidence from other external fertilisers. In Atlantic salmon (*Salmo salar*), Yeates et al. (2013) [7] demonstrated that ovarian fluid mediates cryptic female choice by biasing fertilisation towards conspecific sperm. In Chinook salmon (*Oncorhynchus tshawytscha*), Rosengrave et al. (2016) [13] further showed that ovarian fluid effects on sperm performance translate into differences in fertilisation success and embryo survival. The present results show that comparable FRF-mediated biases can also operate between recently diverged ecotypes within a single species, thereby contributing to reproductive isolation during early ecological divergence. A caveat of the present study is that spermatozoa swimming longevity was assessed visually rather than via computer-assisted sperm analysis (CASA). Because our key inferences about environment- and ecotype-specific longevity differences rest on these visually scored endpoints, the patterns, while compelling, should be interpreted with this constraint in mind. Future CASA-based work could test whether the observed differences reflect sustained progressive motility or earlier reductions in swimming velocity, as these alternatives imply different underlying mechanisms.

Although both female reproductive fluid and ambient water chemistry influence sperm performance, these influences are conceptually distinct. Tests of acid versus basic loch water assess habitat-matching sperm performance and local adaptation to abiotic conditions, a pattern widely reported in external fertilisers [8, 32, 33]. Comparable evidence that FRF can act as a post-mating, pre-zygotic barrier between populations comes from guppies, where FRF differentially affects sperm velocity according to male–female relatedness [15] and population of origin [34]. By contrast, FRF effects test female-derived modulation of sperm behaviour: FRF consistently enhanced sperm longevity beyond that observed in any water control, irrespective of pH, indicating that its effect cannot be explained solely by the physicochemical properties of the spawning environment [5, 16]. Although the two processes may interact under natural spawning conditions, they represent separate layers of post-mating selection. Rather than replacing environmental influences, FRF appears to act as a female-mediated gametic filter, modulating sperm performance within the prevailing abiotic context [7, 35] and providing an additional, biologically structured layer of selection that operates over and above local adaptation to water chemistry [5, 36].

These gametic barriers complement the strong pre-mating isolation previously documented in this system, in which nest architecture and male aggression limit inter-ecotype matings [20]. Importantly, the absence of post-zygotic incompatibilities in in vitro fertilisations [20] indicates that FRF-mediated selection operates at the pre-zygotic stage, potentially acting as an early-acting reinforcing mechanism. By favouring same-ecotype gametes, FRF may bias fertilisation towards same-ecotype gametes and thereby contribute to the maintenance of genomic islands of divergence despite opportunities for gene flow in contact zones [19]. This aligns with theoretical predictions that cryptic female mating bias can accelerate speciation when it targets traits under ecological selection [8, 9].

The mechanisms underlying the observed patterns likely involve both the chemical and physical properties of FRF. In three-spined sticklebacks, the ionic composition of FRF, particularly elevated Na^+^, is the primary factor that prolongs sperm motility from about one minute in freshwater to several hours [23]. Differences in pH, ionic composition, viscosity, and protein content between acid- and base-adapted females may therefore generate ecotype-specific chemoattractant gradients or motility-enhancing conditions for spermatozoa. Future proteomic, metabolomic, and ion-specific analyses of FRF, together with targeted manipulation of candidate molecules, will be needed to identify the precise molecular basis of this assortative response. An additional consideration is that the experiments used wild-caught fish rather than progeny reared under common-garden conditions. Consequently, it remains unclear to what extent the observed differences in FRF function reflect genetically based divergence between the acid and basic ecotypes versus environmentally induced (plastic) differences arising from the contrasting chemical environments experienced by the females during development. Common-garden experiments will be required to disentangle these contributions and to establish the genetic basis of the assortative FRF responses documented here.

## Conclusion

Our results show that female reproductive fluid contributes to gametic isolation between stickleback ecotypes on North Uist by enhancing sperm attraction and longevity in an ecotype- and environment-dependent manner. FRF adds a further, biologically structured layer of post-mating selection beyond local adaptation to water chemistry and represents a previously unrecognised pre-zygotic barrier that can reinforce pre-mating isolation. These findings add to the growing appreciation of non-gametic fluids in sexual selection and highlight the North Uist stickleback radiation as a powerful model for the evolution of multi-stage reproductive barriers during adaptive divergence.

## Electronic supplementary material

Below is the link to the electronic supplementary material.

Supplementary material 1

Supplementary material 2

Supplementary material 3

Supplementary material 4

Supplementary material 5

Supplementary material 6

## Data Availability

Data and R script are available in the FigShare Digital Repository https://doi.org/10.6084/m9.figshare.32593362.
