# Supplementary material for: The role of female reproductive fluid in mediating reproductive isolation between ecotypes of the three-spined stickleback
Source: Zoological Lett. 2026 Sep 22;12:17. doi: 10.1186/s40851-026-00274-7 (PMC13637261; doi:10.1186/s40851-026-00274-7)

Figure S1. A schematic of the sperm selection chamber used. A is the central activation well where sperm are activated. B is the well into which the sperm solution is added. C and D are lateral wells for the chemoattractant (FRF) and the control solution (loch water). Dashed lines indicate the channels connecting the wells.

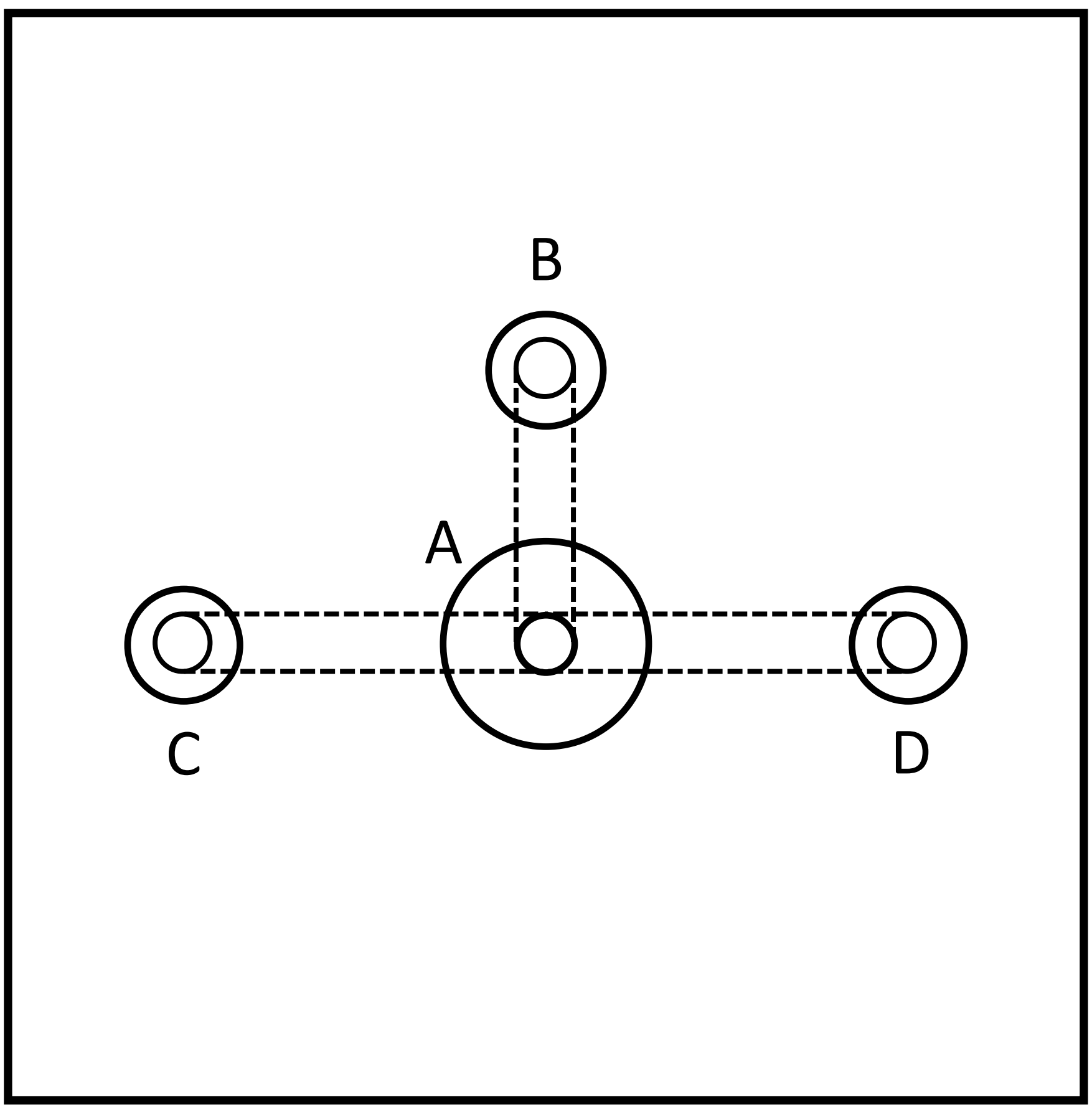

Supplement: Supplementary file 1 [file 40851_2026_274_MOESM6_ESM.pdf]
