# Supplementary material for: The role of female reproductive fluid in mediating reproductive isolation between ecotypes of the three-spined stickleback
Source: Zoological Lett. 2026 Sep 22;12:17. doi: 10.1186/s40851-026-00274-7 (PMC13637261; doi:10.1186/s40851-026-00274-7)
Supplement: Supplementary file 2 [file 40851_2026_274_MOESM1_ESM.docx]

**Table S1.** Final sample sizes by ecotype match and population match. Allopatric diallel (48 trials): same-ecotype/same-population n = 12; same-ecotype/different-population n = 12; different-ecotype/different-population n = 24. Different-ecotype/same-population pairings do not occur in the allopatric diallel because each of those lochs supports a single ecotype. Loch Ciste contact zone (24 trials): same-ecotype n = 12; different-ecotype n = 12; all Ciste pairings are from the same site. SL is Standard Length.

| **Pair** | **Male population** | **Male ecotype** | **Male SL (mm)** | **Female population** | **Female ecotype** | **Female SL (mm)** |
| --- | --- | --- | --- | --- | --- | --- |
| 1 | Bharpa | armourless | 29 | Bharpa | armourless | 40 |
| 2 | Bharpa | armourless | 33 | Bharpa | armourless | 34 |
| 3 | Bharpa | armourless | 30 | Bharpa | armourless | 32 |
| 4 | Bharpa | armourless | 31 | Fada | armourless | 31 |
| 5 | Bharpa | armourless | 29 | Fada | armourless | 31 |
| 6 | Bharpa | armourless | 32 | Fada | armourless | 33 |
| 7 | Bharpa | armourless | 32 | Hosta | armoured | 39 |
| 8 | Bharpa | armourless | 34 | Hosta | armoured | 50 |
| 9 | Bharpa | armourless | 31 | Hosta | armoured | 42 |
| 10 | Bharpa | armourless | 30 | Reivil | armoured | 53 |
| 11 | Bharpa | armourless | 33 | Reivil | armoured | 44 |
| 12 | Bharpa | armourless | 29 | Reivil | armoured | 44 |
| 13 | Fada | armourless | 27 | Bharpa | armourless | 39 |
| 14 | Fada | armourless | 28 | Bharpa | armourless | 32 |
| 15 | Fada | armourless | 32 | Bharpa | armourless | 28 |
| 16 | Fada | armourless | 30 | Fada | armourless | 34 |
| 17 | Fada | armourless | 29 | Fada | armourless | 32 |
| 18 | Fada | armourless | 34 | Fada | armourless | 36 |
| 19 | Fada | armourless | 28 | Hosta | armoured | 36 |
| 20 | Fada | armourless | 28 | Hosta | armoured | 36 |
| 21 | Fada | armourless | 28 | Hosta | armoured | 48 |
| 22 | Fada | armourless | 32 | Reivil | armoured | 39 |
| 23 | Fada | armourless | 32 | Reivil | armoured | 39 |
| 24 | Fada | armourless | 32 | Reivil | armoured | 42 |
| 25 | Hosta | armoured | 42 | Bharpa | armourless | 41 |
| 26 | Hosta | armoured | 46 | Bharpa | armourless | 33 |
| 27 | Hosta | armoured | 36 | Bharpa | armourless | 32 |
| 28 | Hosta | armoured | 40 | Fada | armourless | 32 |
| 29 | Hosta | armoured | 38 | Fada | armourless | 29 |
| 30 | Hosta | armoured | 39 | Fada | armourless | 40 |
| 31 | Hosta | armoured | 39 | Hosta | armoured | 41 |
| 32 | Hosta | armoured | 48 | Hosta | armoured | 52 |
| 33 | Hosta | armoured | 36 | Hosta | armoured | 47 |
| 34 | Hosta | armoured | 43 | Reivil | armoured | 39 |
| 35 | Hosta | armoured | 44 | Reivil | armoured | 49 |
| 36 | Hosta | armoured | 38 | Reivil | armoured | 38 |
| 37 | Reivil | armoured | 38 | Bharpa | armourless | 37 |
| 38 | Reivil | armoured | 41 | Bharpa | armourless | 26 |
| 39 | Reivil | armoured | 38 | Bharpa | armourless | 29 |
| 40 | Reivil | armoured | 43 | Fada | armourless | 33 |
| 41 | Reivil | armoured | 44 | Fada | armourless | 39 |
| 42 | Reivil | armoured | 52 | Fada | armourless | 29 |
| 43 | Reivil | armoured | 48 | Hosta | armoured | 37 |
| 44 | Reivil | armoured | 43 | Hosta | armoured | 47 |
| 45 | Reivil | armoured | 43 | Hosta | armoured | 46 |
| 46 | Reivil | armoured | 41 | Reivil | armoured | 44 |
| 47 | Reivil | armoured | 36 | Reivil | armoured | 43 |
| 48 | Reivil | armoured | 39 | Reivil | armoured | 44 |
| 49 | Ciste | armourless | 42 | Ciste | armourless | 42 |
| 50 | Ciste | armourless | 40 | Ciste | armoured | 42 |
| 51 | Ciste | armourless | 45 | Ciste | armoured | 40 |
| 52 | Ciste | armourless | 39 | Ciste | armourless | 41 |
| 53 | Ciste | armourless | 34 | Ciste | armourless | 48 |
| 54 | Ciste | armourless | 44 | Ciste | armoured | 37 |
| 55 | Ciste | armourless | 46 | Ciste | armourless | 41 |
| 56 | Ciste | armourless | 37 | Ciste | armoured | 50 |
| 57 | Ciste | armourless | 43 | Ciste | armourless | 44 |
| 58 | Ciste | armourless | 44 | Ciste | armoured | 42 |
| 59 | Ciste | armourless | 40 | Ciste | armourless | 43 |
| 60 | Ciste | armourless | 41 | Ciste | armoured | 45 |
| 61 | Ciste | armoured | 38 | Ciste | armoured | 44 |
| 62 | Ciste | armoured | 44 | Ciste | armourless | 39 |
| 63 | Ciste | armoured | 51 | Ciste | armoured | 47 |
| 64 | Ciste | armoured | 46 | Ciste | armourless | 39 |
| 65 | Ciste | armoured | 43 | Ciste | armourless | 41 |
| 66 | Ciste | armoured | 39 | Ciste | armoured | 44 |
| 67 | Ciste | armoured | 40 | Ciste | armourless | 43 |
| 68 | Ciste | armoured | 37 | Ciste | armoured | 45 |
| 69 | Ciste | armoured | 40 | Ciste | armourless | 52 |
| 70 | Ciste | armoured | 42 | Ciste | armoured | 40 |
| 71 | Ciste | armoured | 45 | Ciste | armourless | 54 |
| 72 | Ciste | armoured | 51 | Ciste | armoured | 43 |
