# Supplementary material for: The role of female reproductive fluid in mediating reproductive isolation between ecotypes of the three-spined stickleback
Source: Zoological Lett. 2026 Sep 22;12:17. doi: 10.1186/s40851-026-00274-7 (PMC13637261; doi:10.1186/s40851-026-00274-7)
Supplement: Supplementary file 3 [file 40851_2026_274_MOESM2_ESM.docx]

**Table S2**. Negative binomial GLMM of spermatozoa count as a function of test medium, pair ecotype and population, sperm selection chamber, lateral well, male and female Standard Length and temperature. Sperm stock solution was included as a covariate. Dispersion ratio = 1.62 (P < 0.001). Model random effects: σ^2^ = 0.01; ICC = 0.94, τ_00 Pair_ = 0.13, N _Pair_ = 72. Marginal R^2^ = 0.75, Conditional R^2^ = 0.98, AIC = 2113. IRR is the incidence rate ratio.

| *Coefficient* | *Estimate* | *95% CI* | *IRR* | *95% CI* | *P* |
| --- | --- | --- | --- | --- | --- |
| Intercept | 0.90 | -1.86 – 3.65 | 2.45 | 0.2 – 38.7 | 0.592 |
| Stock count | 0.56 | 0.37 – 0.75 | 1.79 | 1.45 – 2.11 | **<0.001** |
| Medium_(FRF)_ | 0.79 | 0.54 – 1.04 | 2.21 | 1.72 – 2.83 | **<0.001** |
| Ecotype_(same)_ | -0.10 | -0.44 – 0.23 | 0.90 | 0.65 – 1.25 | 0.535 |
| Population_(same)_ | 0.12 | -0.23– 0.47 | 1.13 | 0.80 – 1.60 | 0.495 |
| Temperature | -0.01 | -0.12– 0.12 | 1.00 | 0.89 – 1.13 | 0.990 |
| Chamber_(two)_ | 0.11 | -0.12 – 0.33 | 1.11 | 0.89 – 1.40 | 0.360 |
| Well_(D)_ | -0.05 | -0.02 – 0.18 | 0.95 | 0.76 – 1.20 | 0.688 |
| Male SL | 0.01 | -0.02 – 0.02 | 1.00 | 0.98 – 1.02 | 0.871 |
| Female SL | -0.01 | -0.02 – 0.02 | 1.00 | 0.98 – 1.02 | 0.867 |
| Medium_(FRF)_:Ecotype_(same)_ | 0.50 | 0.18 – 0.82 | 1.65 | 1.20 – 2.27 | **0.002** |
| Medium_(FRF)_:Population _(same)_ | -0.18 | -0.50 – 0.13 | 0.83 | 0.61 – 1.14 | 0.259 |
