# Supplementary material for: The role of female reproductive fluid in mediating reproductive isolation between ecotypes of the three-spined stickleback
Source: Zoological Lett. 2026 Sep 22;12:17. doi: 10.1186/s40851-026-00274-7 (PMC13637261; doi:10.1186/s40851-026-00274-7)
Supplement: Supplementary file 4 [file 40851_2026_274_MOESM3_ESM.docx]

**Table S3**. Negative binomial GLMM of spermatozoa motility duration as a function of test medium, temperature, pair ecotype, male environment of origin, and male and female Standard Length. Sperm stock solution was included as a covariate. Dispersion ratio = 1.28 (P <0.001). Model random effects: σ^2^ = 0.02; ICC = 0.80, τ_00 Pair_ = 0.05, N _Pair_ = 72. Marginal R^2^ = 0.96, Conditional R^2^ = 0.99, AIC = 8480. IRR is the incidence rate ratio. Pairwise Šidák post-hoc contrasts showed highly significant differences (P <0.001) in spermatozoa longevity among media in the order: 3-spine FRF > 9-spine FRF > basic loch water > acid loch water > fishless loch water/distilled water. There was no significant difference in longevity between fishless loch water and distilled water (P = 0.999).

| *Coefficient* | *Estimate* | *95% CI* | *IRR* | *95% CI* | *P* |
| --- | --- | --- | --- | --- | --- |
| Intercept | 9.22 | 7.64 – 10.80 | 10101 | 2084 – 48963 | **<0.001** |
| Log_10_ stock count | -0.16 | -0.27 – -0.05 | 0.85 | 0.77 – 0.95 | **0.005** |
| Temperature | -0.03 | -0.09 – 0.04 | 0.97 | 0.91 – 1.04 | 0.374 |
| Ecotype_(same)_ | 0.04 | -0.08 – 0.16 | 1.04 | 0.92 – 1.17 | 0.524 |
| Male environment_(basic)_ | 0.05 | -0.12 – 0.22 | 1.05 | 0.89 – 1.25 | 0.566 |
| Medium_(9-spine FRF)_ | -0.11 | -0.18 – -0.04 | 0.90 | 0.83 – 0.96 | **0.003** |
| Medium_(basic lochs)_ | -0.54 | -0.60 – -0.47 | 0.59 | 0.55 – 0.63 | **<0.001** |
| Medium_(acid lochs)_ | -1.88 | -1.99 – -1.78 | 0.15 | 0.14 – 0.17 | **<0.001** |
| Medium_(fishless lochs)_ | -3.00 | -3.16 – -2.85 | 0.05 | 0.04 – 0.06 | **<0.001** |
| Medium_(distilled water)_ | -3.10 | -3.32 – -2.88 | 0.05 | 0.04 – 0.06 | **<0.001** |
| Male SL | 0.01 | -0.01 – 0.02 | 1.01 | 0.99 – 1.02 | 0.302 |
| Female SL | 0.01 | -0.01 – 0.02 | 1.01 | 1.00 – 1.02 | 0.172 |
| Male environment_(basic)_: Medium_(9-spine FRF)_ | 0.01 | -0.09 – 0.11 | 1.01 | 0.91 – 1.12 | 0.865 |
| Male environment_(basic)_: Medium_(basic lochs)_ | 0.17 | 0.07 – 0.26 | 1.18 | 1.08 – 1.30 | **<0.001** |
| Male environment_(basic)_: Medium_(acid lochs)_ | -0.62 | -0.78 – -0.46 | 0.54 | 0.46 – 0.63 | **<0.001** |
| Male environment_(basic)_: Medium_(fishless lochs)_ | -0.29 | -0.51 – -0.07 | 0.75 | 0.60 – 0.94 | **0.011** |
| Male environment_(basic)_: Medium_(distilled water)_ | 0.03 | -0.26 – 0.33 | 1.04 | 0.77 – 1.39 | 0.820 |
