# Supplementary material for: The role of female reproductive fluid in mediating reproductive isolation between ecotypes of the three-spined stickleback
Source: Zoological Lett. 2026 Sep 22;12:17. doi: 10.1186/s40851-026-00274-7 (PMC13637261; doi:10.1186/s40851-026-00274-7)
Supplement: Supplementary file 5 [file 40851_2026_274_MOESM4_ESM.docx]

**Table S4**. Negative binomial GLMM of spermatozoa motility duration as a function of test medium, temperature, pair ecotype, male environment of origin for acid and basic loch water only, and male and female Standard Length. Sperm stock solution was included as a covariate. Dispersion ratio = 1.22 (P = 0.080). Model random effects: σ^2^ = 0.03; ICC = 0.71, τ_00 Pair_ = 0.07, N _Pair_ = 48. Marginal R^2^ = 0.91, Conditional R^2^ = 0.97, AIC = 2514. IRR is the incidence rate ratio.

| *Coefficient* | *Estimate* | *95% CI* | *IRR* | *95% CI* | *P* |
| --- | --- | --- | --- | --- | --- |
| Intercept | 9.29 | 7.14– 11.44 | 10818 | 1260 – 92904 | **<0.001** |
| Log_10_ stock count | -0.14 | -0.28 – -0.01 | 0.87 | 0.75 – 1.00 | **0.042** |
| Temperature | -0.07 | -0.16 – 0.02 | 0.93 | 0.86 – 1.02 | 0.132 |
| Ecotype_(same)_ | -0.04 | -0.21 – 0.13 | 0.96 | 0.81 – 1.13 | 0.626 |
| Male environment_(basic)_ | 0.16 | -0.16 – 0.48 | 1.17 | 0.85 – 1.61 | 0.341 |
| Medium_(acid lochs)_ | -1.32 | -1.42 – -1.22 | 0.27 | 0.24 – 0.29 | **<0.001** |
| Male SL | 0.01 | -0.02 – 0.03 | 1.01 | 0.98 – 1.03 | 0.609 |
| Female SL | 0.00 | -0.01 – 0.02 | 1.00 | 0.99 – 1.02 | 0.611 |
| Male environment_(basic)_: Medium_(acid lochs)_ | -0.95 | -1.11 – -0.79 | 0.39 | 0.33– 0.45 | **<0.001** |
