# Supplementary material for: The role of female reproductive fluid in mediating reproductive isolation between ecotypes of the three-spined stickleback
Source: Zoological Lett. 2026 Sep 22;12:17. doi: 10.1186/s40851-026-00274-7 (PMC13637261; doi:10.1186/s40851-026-00274-7)
Supplement: Supplementary file 6 [file 40851_2026_274_MOESM5_ESM.docx]

**Table S5**. Negative binomial GLMM of spermatozoa motility duration as a function of test medium, temperature, pair ecotype, male environment of origin for acid and basic water (Loch Ciste only), and male and female Standard Length. Sperm stock solution was included as a covariate. Dispersion ratio = 1.46 (P = 0.08). Model random effects: σ^2^ = 0.02; ICC = 0.56, τ_00 Pair_ = 0.03, N _Pair_ = 24. Marginal R^2^ = 0.89, Conditional R^2^ = 0.95, AIC = 672. IRR is the incidence rate ratio.

| *Coefficient* | *Estimate* | *95% CI* | *IRR* | *95% CI* | *P* |
| --- | --- | --- | --- | --- | --- |
| Intercept | 6.35 | 3.01 – 9.69 | 572 | 20 – 16197 | **<0.001** |
| Log_10_ stock count | 0.12 | -0.10 – 0.35 | 1.13 | 0.91 – 1.41 | 0.274 |
| Temperature | 0.05 | -0.07 – 0.17 | 1.05 | 0.93 – 1.18 | 0.434 |
| Ecotype_(same)_ | 0.10 | -0.09 – 0.30 | 1.11 | 0.92 – 1.34 | 0.284 |
| Male environment_(basic)_ | 0.13 | -0.12 – 0.37 | 1.13 | 0.89 – 1.44 | 0.308 |
| Medium_(acid water)_ | -1.08 | -1.29 – -0.86 | 0.34 | 0.28 – 0.42 | **<0.001** |
| Male SL | -0.01 | -0.03 – 0.02 | 0.99 | 0.97 – 1.01 | 0.509 |
| Female SL | -0.02 | -0.04 – 0.01 | 0.98 | 0.96 – 1.01 | 0.222 |
| Male environment_(basic)_: Medium_(acid water)_ | -0.39 | -0.70– -0.08 | 0.68 | 0.50– 0.92 | **0.013** |
